# Nothing changes if nothing changes

**DOI:** 10.1093/icvts/ivac217

**Published:** 2022-08-18

**Authors:** Michael Salna, Brett R Anderson, Emile Bacha, Paul Kurlansky

**Affiliations:** Division of Cardiac Surgery, Columbia University Irving Medical Center, New York, NY, USA; Department of Pediatrics, Columbia University Irving Medical Center, New York, NY, USA; Division of Cardiac Surgery, Columbia University Irving Medical Center, New York, NY, USA; Division of Cardiac Surgery, Columbia University Irving Medical Center, New York, NY, USA; Center for Innovation and Outcomes Research, Columbia University Irving Medical Center, New York, NY, USA

**Keywords:** Surgical education, Simulation, Resident education, Surgery training

A musician would not play a concert piece without repeatedly practicing each measure flawlessly. Similarly, the first time a professional basketball player takes a three-pointer is not during a televised playoff game. That shot is taken after countless iterations of micro-improvements in their stance, jump, and wrist motion on the practice court. These performance-based professionals practice until their default is near perfection, and then they continue to be coached throughout their professional career. With an arguably steeper learning curve, why are surgeons not afforded this luxury of preparation and ongoing mentorship? The clock cannot be stopped in the operating room and, unlike hitting a wrong note on the piano, every misplaced stitch or cut may have irreversible consequences, which may not be apparent at the time.

Learning cardiac surgery is stressful. The stakes are high, cross-clamp and bypss times are precious and the cognitive burden can be immense. To further complicate matters, as outcome measures become increasingly scrutinized and operative costs rise in the face of declining reimbursement, stress falls not only upon the trainee but upon the attending surgeon as well. Despite these rigours, cardiac surgery is still fundamentally taught within a mentor-mentee apprenticeship training model that largely ends after fellowship. It may be more sophisticated nowadays, but a cardiac surgeon teaches residents the same way a violin master would teach an apprentice to build a violin in the 15th-century Florence or a stone mason an apprentice during the building of a great cathedral. Why has it not changed?

Every case is a playoff game for surgeons. Every day we must perform technically and physically demanding tasks, aspiring to nothing short of excellence. Nathan *et al.* [1] previously demonstrated that technical performance in paediatric cardiac surgery was strongly associated with outcomes—to the point where optimal technical performance can overcome adverse intraoperative events. By extension, poor performance is associated with short- and long-term mortality and reintervention [2, 3]. So, if technique is so important, surely there are objective measures to assess technical performance in trainees?

Hussein *et al.* performed a systematic review of 54 studies evaluating the use of competency-based assessments in the evaluation of technical skills in cardiothoracic surgery. Cardiac surgery was the most common specialty using objective assessment methods with coronary anastomosis being the most frequently tested task (28%). Thirty studies (56%) assessed objective changes in technical performance (the others validated the assessment tools) and 97% of them found improvement in their trainees. Despite this obvious benefit, it was surprising that only 21 (39%) of the 54 studies incorporated assessment methods into their training curricula. Clearly, there is a mismatch between our acknowledgement of the importance of simulation and technical preparation and its actual implementation into training and ongoing career development.

This is not for the lack of trying. Numerous studies have been published on innovative training tools and curricula—ranging from bootcamps [4] to porcine hearts [5] to 3D-printed models [6]. These then raise the questions of—which of these translate into real operative improvement? Who will pay for them? And, as Hussein *et al.* bring up, who is the best person to proctor simulation? It is not enough for programs to simply implement simulation programs because not all practice and simulation is made equal. This also makes measuring of their effectiveness in a meta-analysis very difficult.

There is no substitute for learning in the operating room. Here, trainees are challenged to not only develop technical skills but also critical thinking, complex decision-making, and judgement—equally important qualities that can only be honed from clinical experience. However, there are a myriad of factors liming this exposure: work hour restrictions, regulatory scrutiny limiting autonomy, hospital pressures for greater efficiency and reduction in straightforward procedures as patient complexity increases and minimally invasive options are popularized [7]—not to mention the ever-present risk to patient outcome inherent in trainee learning curves.

Therefore, as the external learning environment evolves, so too should our specialty. Pilots log hundreds of hours virtually flying through inclement weather and troubleshooting device malfunctions before captaining their own planes. Why should surgeons not benefit from such a training and assessment paradigm? The integration of simulation and technical performance testing into training programs and ongoing career development may accelerate technical learning and thereby enhance learning in the operating room—both the technical and non-technical.

In 2013, in a landmark study, Birkmeyer *et al.* [8], 20 attending bariatric surgeons in Michigan videotaped themselves operating, rated each other’s technical skill, and found strong associations between technical skill and patient postoperative complications and mortality. As a result, in 2014, the American Board of Colon and Rectal Surgery included a version of the Objective Structured Assessment of Technical Skill as a mandatory component of their certification [9].

The late James Tweddell advocated for the addition of technical performance examinations in congenital heart surgeons—whether by standardized skill stations, direct observation or submission of videos [10]—and participated in and helped direct the ongoing Congenital Heart Technical Skill Study, assessing associations attending congenital heart technical skill and patient outcomes [11]. Perhaps the inclusion of a practical examination component by the ABTS will hone our attention into optimizing objective assessment measures and thereby enhancing our training of the next generation of excellent cardiothoracic surgeons.

The unanswered question which undoubtedly underlies the surprising reluctance to incorporate simulation into training programs exposed by Hussein and colleagues is: Do we have the right tools? Is there convincing evidence that current simulation techniques actually translate into improved operative performance for cardiac surgery. Future research clearly needs to focus on the answer to this question. Otherwise, nothing will change if we do not change.
